# Are there risk factors commonly observed on Australian farms where the welfare of livestock is poor?

**DOI:** 10.1017/awf.2024.27

**Published:** 2024-09-16

**Authors:** Natarsha Williams, Lauren Hemsworth, Sarah Chaplin, Richard Shephard, Andrew Fisher

**Affiliations:** 1 Animal Welfare Science Centre, Faculty of Science, University of Melbourne, Parkville, VIC 3010, Australia; 2 Agriculture Victoria, Department of Energy, Environment and Climate Action, Tatura, VIC 3616, Australia; 3 School of Electrical and Data Engineering, Faculty of Engineering & IT, University of Technology, Sydney, NSW, Australia

**Keywords:** Animal welfare, cattle, farmers, goats, livestock, sheep

## Abstract

The objective of this study was to identify factors more commonly observed on farms with poor livestock welfare compared to farms with good welfare. Potentially, these factors may be used to develop an animal welfare risk assessment tool (AWRAT) that could be used to identify livestock at risk of poor welfare. Identifying livestock at risk of poor welfare would facilitate early intervention and improve strategies to promptly resolve welfare issues. This study focuses on cattle, sheep and goats in non-dairy extensive farming systems in Australia. To assist with identifying potential risk factors, a survey was developed presenting 99 factors about the farm, farmers, animals and various aspects of management. Based on their experience, key stakeholders, including veterinarians, stock agents, consultants, extension and animal welfare officers were asked to consider a farm where the welfare of the livestock was either high or low and rate the likelihood of observing these factors. Of the 141 responses, 65% were for farms with low welfare. Only 6% of factors had ratings that were not significantly different between high and low welfare surveys, and these were not considered further. Factors from poor welfare surveys with median ratings in the lowest 25% were considered potential risks (n = 49). Considering correlation, ease of verification and the different livestock farming systems in Australia, 18 risk factors relating to farm infrastructure, nutrition, treatment and husbandry were selected. The AWRAT requires validation in future studies.

## Introduction

Incidents of poor livestock welfare that are non-compliant with the relevant animal welfare legislation or standards, are an ongoing problem (Kelly *et al.* 2011; Lomellini-Dereclenne *et al.* 2017; Grandin 2018; Hedman *et al.* 2018; Williams *et al.* 2024), and the focus of this study. The majority of livestock welfare non-compliance results from a chronic failing to provide the animals’ basic needs rather than a malicious act (Sentencing Advisory Council [SAC] 2019; Williams *et al.* 2024). Examples include inadequate treatment, poor environment, unsuitable nutrition, (Hedman *et al.* 2018; Temple & Manteca 2020; Väärikkälä *et al.* 2020; Williams *et al.* 2024) or improper management and husbandry (Williams *et al.* 2024). Small and large landholders with stock numbers from tens to thousands have been identified with welfare non-compliance (Kelly *et al.* 2011; Hedman *et al.* 2018; Väärikkälä *et al.* 2020; Williams *et al.* 2024). Notwithstanding this variety of farm settings, anecdotally, situations where livestock welfare is identified as being non-compliant are predictable. There are factors that are typically observed during farm visits where the welfare of the livestock is poor (Williams 2024) and these may be considered as risk factors.

A risk factor can be any characteristic about an individual, their environment or situation (Australian Psychological Society 2023) that is statistically correlated with a problem outcome, but without necessarily being a cause of the problem (O’Connell *et al.* 2009; Garrett & Monahan 2019). On occasion, risk factors might be predictors and also a direct cause of the problem (Schooling & Jones 2018; Garrett & Monahan 2019), while some factors are causal but not predictors (Schooling & Jones 2018). Together, a range/combination of risk factors can be used to perform a risk assessment, to predict a variety of outcomes (Desmarais *et al.* 2012; Garrett & Monahan 2019). An animal welfare risk assessment tool (AWRAT) could potentially be used to identify livestock at risk of poor welfare. This may facilitate early intervention, extension and education and more timely and sustained resolution of livestock welfare issues. In contrast, protective factors are characteristics associated with a reduced risk of a negative outcome (O’Connell *et al.* 2009). In the context of animal welfare, risk and protective factors may include anything to do with the farm, farmer, animals, nutrition, management and husbandry.

Risk assessment is already used to predict outcomes in a range of situations, including child protection (D’Andrade *et al.* 2008), family violence (Desmarais *et al.* 2012; Victoria Police 2019) and substance abuse (Stone *et al.* 2012; van der Put *et al.* 2013). Risk assessment in these contexts identifies high and low risk offenders and reoffenders (van Ginneken 2019). In addition, researchers in Denmark and Sweden have tried to use pre-recorded information in established data registers to predict poor welfare in dairy herds (Sandgren *et al.* 2009; Houe *et al.* 2011; Nielsen 2011; Nyman *et al.* 2011; Otten *et al.* 2014). Initial trials showed some predictive capacity, but all studies indicated further work was required before they could be implemented as reliable tools (Sandgren *et al.* 2009; Houe *et al.* 2011; Nyman *et al.* 2011; Otten *et al.* 2014). In the Republic of Ireland, key performance indicators based on national data-sets, for example, late registrations and on-farm burial, were unsuccessful at predicting poor welfare in dairy and beef cattle, sheep and horses (Kelly *et al.* 2011, 2013). One of the main barriers to using pre-recorded data to measure risk of poor welfare in extensive farming systems in Australia is the absence of accessible relevant herd records. Other than livestock movements and notifiable diseases, the majority of health recording is performed at the farm level.

Literature focused upon identifying risk factors associated with situations of poor livestock welfare in Australia are rare. Some international studies have focused upon the human element of livestock welfare incidents, and have identified farmer-related challenges including: financial issues (Andrade & Anneberg 2014; Devitt *et al.* 2015; Farm Animal Welfare Council [FAWC] 2016), age-based limitations (Devitt *et al.* 2015), lack of qualification (Lomellini-Dereclenne *et al.* 2017) and mental (Andrade & Anneberg 2014; Devitt *et al.* 2015; FAWC 2016) and physical health problems (FAWC 2016). Studies from the European Union (EU) and United Kingdom (UK) have identified issues that occurred on properties where welfare non-compliance has been found. These include a failure to provide: sick and injured animals with adequate treatment (Otten *et al.* 2014; Lomellini-Dereclenne *et al.* 2017; Väärikkälä *et al.* 2020), adequate housing (Lomellini-Dereclenne *et al.* 2017; Hedman *et al.* 2018; Väärikkälä *et al.* 2020), infrastructure (Lomellini-Dereclenne *et al.* 2017), nutrition (Lomellini-Dereclenne *et al.* 2017; Väärikkälä *et al.* 2020), appropriate record-keeping (Lomellini-Dereclenne *et al.* 2017), increased livestock mortality (Sandgren *et al.* 2009; Kelly *et al.* 2011), reluctance to engage a veterinarian (Lomellini-Dereclenne *et al.* 2017) and small herd size (Väärikkälä *et al.* 2019). Potentially, any or all of these factors could be predictors of livestock welfare non-compliance. Notably, however, some of these studies were focused upon dairy farms and all were based in the EU and UK where farming tends to be more intensive with indoor housing in some areas, compared to Australia’s extensive systems.

In a companion study by the authors (published simultaneously; Williams *et al.* 2024), 39 years of historical animal welfare investigation records, from Victoria, Australia were reviewed. The presence or absence of more than 60 factors were recorded for each case. As the data had few set fields and were not produced with the intention of detailed analysis, the presence/absence of each factor could not be determined in every case. The issues and characteristics most commonly observed included animals that were in poor body condition, overstocked, unwell, injured, recumbent or deceased. Other factors included a failure to wean, cull, draft, feed or adequately supervise stock, and farmers that were unreliable at doing what they had been asked or said they would do (Williams *et al.* 2024).

Further consultations with veterinarians, stock agents, farm consultants and animal welfare officers in future work by the authors (Williams 2024) also identified issues and characteristics frequently observed on Australian farms where the welfare of the animals was poor. These included farms that were untidy and run-down or had poor infrastructure, pulled wool on fences and sheep, scouring livestock, poor quality hay, little pasture compared to the local area, a failure to castrate male stock, year-round joining, small land holders or the presence of a considerable range of animals (Williams 2024). A number of issues and characteristics related to the farmer were also identified during these consultations, including: mental and physical health issues; a lack of time; relationship issues or disputes; a lack of support or knowledge; absenteeism; and, finally, farmers that were disengaged or showed a poor attitude towards farming (Williams 2024).

This study aimed to identify risk factors that were more commonly observed on farms where there is low livestock welfare compared to farms where the welfare is high. A survey of key stakeholders was used to identify possible risk and protective factors that were more commonly observed on farms with low and high livestock welfare, respectively. Stakeholders surveyed included animal health and extension officers, veterinarians, stock agents and private consultants. Some participants were likely to have experience in farms with good welfare (e.g. extension officers and consultants) and others poor welfare (animal health officers). Participants from different locations were expected to have experience in different production systems, providing an opportunity to consider a diverse range of views. In addition, surveying farms with high and low livestock welfare would allow the presence/absence of the factors to be compared between farms with different welfare standards. The intention was for the risk factors identified during this study to be included in a proposed animal welfare risk assessment tool (AWRAT). The proposed AWRAT could potentially be used by agencies that investigate livestock welfare non-compliance issues on-farm to identify livestock at risk of poor welfare. This may assist with planning and resourcing the investigation, potentially leading to faster and sustained resolution of welfare issues. Ideally, the tool would include less than 20 factors, so it could be completed easily by inspectors or officers responding to instances of poor welfare. In this study, livestock refers to cattle, sheep and goats kept in non-dairy, extensive farming systems in Australia.

## Materials and methods

### Ethical approval

This project has human research ethics approval from The University of Melbourne; ID:20808. The data-set presented in this paper are not readily available because our ethics approval specifies: ‘*Data are aggregated and analysed and reported as a group, therefore no findings that could identify any individual will be published.*’ Therefore, we cannot supply raw data even though they are anonymised, without special approval from the ethics committee through an amendment. Requests to access the datasets should be directed to NW (natscottw@gmail.com).

### Survey

#### Survey structure

A survey was developed with four sections: an introduction, questions about the participant and two main survey sections. The introduction included the eligibility criteria, the aim of the survey, support service contacts and key definitions. The World Organisation of Animal Health (WOAH) definition of animal welfare was included, providing guidance as to the intended meaning of animal welfare in this research (WOAH 2023). The participant details collected consisted of: work role, experience, post code and an opportunity to provide contact details for future discussion with the researcher. This paper relates to the section of the survey titled: ‘Identifying factors that might be observed on a property where there is a high/low standard of livestock welfare?’ The subsequent section titled ‘What are the issues and challenges that livestock farmers face? How can farmers be supported to ensure they are able to provide suitable care, management and welfare of their animals?’ will be reported in a future paper (Williams 2024). A comprehensive list of 99 potential risk factors was generated, based upon information from a number of sources, including consultations with industry (Williams 2024), a review of past animal welfare investigations from 1981–2020 (Williams *et al.* 2024) and reviews of the literature (Williams 2024). Factors were selected for inclusion if they were relevant to non-dairy cattle, sheep and goat extensive production systems in Australia, easily observable on a routine farm visit without consultation with the farmer and not discriminatory to minority groups (Tonry 2014). The 99 potential risk factors included 16 about the farm including the standard of the stock-handling facilities, fences and water infrastructure. There were 13 nutrition factors covering the quality, quantity and appropriateness of feed for the types of animals present. The 14 factors about husbandry and management included the timeliness of joining, weaning, marking, crutching, culling, drenching and shearing. The body condition score, presence of lameness, scouring or lice are examples of the 14 factors about animals. Lastly, the 33 factors about the farmer included challenges with: mental and physical health, age, relationships, lack of support, available time, knowledge and attitude. The complete list of factors included in the survey are listed by section in the Supplementary material.

There was one version of this survey section, but it was completed in either of two ways: considering a farm with high or low welfare. The farm welfare standard, high/low for each participant, was determined by the researchers, as outlined below.

The draft survey was trialled by six participants with livestock and farming knowledge to check for errors and clarity. Only minor edits were required. The survey was open from October 2021 to March 2022 and recruitment of participants was staggered over that period.

#### Participant recruitment

The survey sampled participants from the following occupations: veterinarians, stock agents, private farm consultants, extension and animal welfare officers. To be eligible, participants needed to be more than 18 years old and have more than six months’ experience working with non-dairy cattle, sheep or goats. A minimum of six months’ experience was chosen to ensure participants had some relevant knowledge of livestock farming but also allowing for opinions of those new to the industry that may have more contemporary views on livestock welfare. Convenience sampling was used to recruit participants for the survey, as a representative sample of the industry was not necessary for the purpose of identifying potential risk factors.

All recruitment was completed via email. An initial engagement email was sent to each contact and then a follow-up email sent no less than nine days later. All contact details (email addresses) were sourced from the public domain and accessed using an internet search engine or were contacts of NW. Sharing access to the survey (i.e. snowball recruitment) to similarly qualified potential participants was encouraged. Engagement emails were sent to the Australian Veterinary Association (AVA) who forwarded them on to the Australian Cattle Veterinarian Group and the Sheep, Camelid and Goat Veterinarians groups for dissemination to their members at their discretion. Engagement emails were sent directly to 182 veterinary clinics and 12 individual veterinary practitioners that worked with livestock (based on available information). To engage stock agents, the Australian Livestock and Property Agents Association (ALPA) were contacted first, then seven national and 330 local stock agencies and 2,145 individual stock agents were sent the engagement email. Private farm consultants were contacted individually. Government animal welfare departments in all states and Territories and the Royal Society for Prevention of Cruelty to Animals (RSPCA) in New South Wales and Victoria were asked to disseminate the engagement material to their animal welfare officers/inspectors to compete the survey. To engage extension officers, emails were sent to various extension groups, for example, Best Wool Best Lamb and Future Beef. A further 128 individual extension officers were contacted directly.

The survey was available through the Qualtrics survey platform (Qualtrics, Provo, UT, USA) and was able to be accessed using a mobile telephone, tablet or computer via a QR code or direct link. Participation in the survey was voluntary and anonymous. Using a visual analogue slider scale, participants were asked to rate how true or relevant they felt each of the 99 factors was in respect to a situation with either a high or low livestock welfare standard. Occupation and likely experience were used to determine whether participants completed the survey for either farms with high or low welfare. The non-random allocation of most participants to either high or low welfare farm surveys was to ensure the study benefited from the most informed opinions. Animal welfare officers (AWO) were asked to complete the survey for farms with low welfare (LWFS) as this is an important part of their role. Consultants and extension officers often work with farmers where the welfare standard is higher and so were asked to complete the high welfare farm survey (HWFS). Stock agents and veterinarians were allocated either high or low welfare, (based on the month of their birth) as they are likely to have experience with farms with a range of welfare standards. Those born on odd months were allocated low welfare and even months, high welfare farms. All participants were asked to consider only properties where there were at least ten livestock of the relevant type (non-dairy cattle, sheep or goats). The slider scale had no numbers, and the scale extremes were marked ‘never true’ on the left which was equivalent to 0 and ‘always true’ on the far right which was equivalent to a rating of 100 and the pointer was initially placed in the middle of the sliding scale. All questions had an option to tick a box ‘I don’t know’. The point that participants selected as their rating on the slider scale was converted to a number from 0–100 by the survey platform. Participants could exit the survey at any point and incomplete submissions remained open for two weeks before being closed automatically.

#### Survey statistical analysis

The data analysis focused on identifying risk factors that were most likely to be observed on properties where the welfare of the livestock was poor (LWFS) compared to other farms. Responses to the HWFS were analysed more broadly in the current paper and will be analysed further in a future work (Williams 2024). LWFS and HWFS responses were downloaded and analysed in Microsoft Excel®. All surveys with at least one response were included in the analysis.

The proportion of participants from different locations, occupations and with varying experience were calculated to determine the diversity of participants in the survey. A Chi-squared test was used to compare the proportion of responses based on a number of variables within the study. Values were considered to be significantly different when *P* < 0.05. Factors that had not been rated and those that had been ticked ‘I don’t know’ were combined into a single category of ‘no response’ (NR) for analysis.

For LWFS data, the Kruskal-Wallis test was used to compare the responses for participants based on their occupation and then their location in either the north or south of Australia. The occupations compared were veterinarians, stock agents, animal welfare officers and ‘other’. The group ‘other’ included consultants, rural retailers, a farm manager and a farm hand. The north region of Australia included Queensland (QLD), Northern Territory (NT), and in Western Australia (WA) any participants located north of the level of the South Australian (SA)/NT border. Conversely, the south region of Australia included Victoria, Tasmania, New South Wales, South Australia and Western Australia south of the level of the northern SA border.

A Krukal-Wallis test was used to compare the ratings in HFWS and LFWS responses for each factor. Using the individual sliding scale scores, a median rating for each factor for both survey types was calculated. Median was preferred as it was less likely to be impacted by extreme outlier ratings. Median ratings of < 26 or > 74 were considered in the key range.

A Chi-squared test was used for pair-wise comparison of the factors that had not been excluded in the previous steps. The ordinal scores for each of the variables were collapsed into a dichotomy of counts of scores ≤ 26 and > 26. The distribution of these counts underwent collation for pairs of variables using a two-by-two table. Differences between the distributions of the two variables were examined using a Chi-squared test with one degree of freedom. Pairs of factors were considered not to differ when *P* ≥ 0.8.

### Selection of risk factors for the Animal Welfare Risk Assessment Tool (AWRAT)

The aim was to develop an AWRAT that included risk factors that would be correlated with poor welfare outcomes, be short enough to be a feasible tool and include factors that demonstrated a logical connection to poor welfare outcomes. It was also the intent to ensure that the risk factors were agreed upon from a range of industry perspectives and applicable to the array of different production systems seen in Australia. The process to select risk factors for the proposed AWRAT, was assisted by considering the stakeholders’ views provided in the survey, personal experience, discussions with industry in a previous study and a number of other practical considerations. The details of each step and the reasoning are as follows:

1. Only factors with ratings that were significantly different (*P* ≤ 0.05) between HWFS and LWFS responses were considered potential risk factors. This was to ensure that the factors were more likely to differentiate farms with low welfare standards.
2. Only factors in the LFWS with medians in the key range (< 26) were considered further. This range was selected as ratings in the bottom 25% of the scale were more likely to reflect a strong opinion that the factor was present/absent on low welfare farms.
3. Factors that had ratings that were not significantly different between participants from the various occupations and comparing those from the north and south regions of Australia were preferentially selected to be included in the proposed AWRAT. This was to ensure that participants offering different industry perspectives from different production systems largely agreed upon the likelihood of these factors being observed on farms where the welfare of the livestock was poor.
4. To reduce the number of factors further, factors that were found to have a very similar likelihood of being found on farms with poor welfare according to survey participants, were identified. Those pairs of variables that did not differ in their dichotomised scores (*P* ≥ 0.8) were considered to be measuring the same (hidden) factor impacting on welfare outcomes (i.e. the scores are correlated). Therefore, only one of the correlated pair of variables was required to describe the effect and therefore only one was included in the AWRAT. Therefore, retaining just one of each pair of correlated factors was unlikely to reduce the robustness of the tool to assess risk. Factors were preferentially retained if they were: easy to observe and verify during farm visits, relevant to all livestock farming systems in Australia, clear to minimise ambiguity and notably different to the other factors already selected in the AWRAT.

## Results

### Survey participants

Overall, 141 at least partially completed responses were received, the majority of responses were for LFWS (n = 91) compared to HFWS (n = 50). No one factor had a rating from all participants. It was not possible to calculate the response rate for the survey as many of the recruitment emails were sent to businesses rather than to individuals and sharing was encouraged with suitably experienced colleagues. However, considering the number of responses received and the number of engagement emails sent, the response rate was low.

From the 141 survey responses, almost half of all respondents were AWO (47%). The remainder included stock agents (21%), veterinarians (11%), private consultants (10%) and extension officers (6%). There were survey responses from a rural retailer, farm manager and farm hand, who were not strictly targets of this study but were retained as they met the other criteria for participation. The vast majority of participants (92%) had more than five years’ experience working with livestock. Most participants had experience with cattle (91%) and/or sheep (88%) and 50% had experience with goats. Almost 20% of participants were from the north region of Australia and 81% from the south.

### Survey – factors analysis

Six factors had ratings that did not differ significantly between HWFS and LWFS responses (*P* > 0.05). These factors were: farms with more than one enterprise (A.7), included more than one block of land (A.10) or ran breeding livestock (C.2); and farmers that worked off-farm (E.17), had significant off-farm commitments (E.18) or had multiple properties (E.30). All were excluded from the list of risk factors for further consideration from the proposed AWRAT. Fifty of the factors in the LWFS (51%) did not have medians in the key range, were not considered to be reliable predictors of poor livestock welfare and were therefore excluded from the proposed AWRAT. This included 82% of factors about the farmer, 38% of farm factors, 15% of nutrition-related factors, 35% of management factors and 50% of animal factors. A further three factors had median ratings that were significantly different between the north and south regions of Australia and were removed. Table 1 summarises how 81 factors were removed from the initial 99, leaving 18 factors that were included in the proposed AWRAT.

**Table 1.** Step-wise process to exclude 81 factors from the initial list of 99, to develop the proposed AWRAT. The factors are listed by their individual identifying code. The entire list of factors and their codes are in the supplementary material

Factors removed	Justification for exclusion from proposed AWRAT
A.7, A.10, D.2, E.17, E.18, E.30	No significant difference between ratings for factors for HWFS and LWFS (*P* > 0.05)
A.3, A.11, A.12, A.15, B.2, B.3, C.3, C.5, C.6, C.13, C.15, C.18, C.21, C.23, D.1, D.4, D.5, D.7, D.8, D.12, E.3, E.5–11, E.13–16, E.19, E.21–29, E.31, E.32	Median rating outside the key range for LWFS
D.11, D.14, E.1	Ratings were significantly different between the north and south regions of Australia (*P* ≤ 0.05)
A.14	Add ‘accidental injury’ to B.7, remove A.14
C.2	Difficult to verify, correlated with A.1
E.12	Difficult to verify, correlated with B.7
C.14, D.13	Only relevant to farms with sheep. No sheep in NT and limited sheep in QLD
D.6	Relying on officer’s familiarity with condition scoring, correlated to B.7
E.2, E.20	Both subjective, difficult to verify, correlated with factors E.2 – A.5, E.20 –C.1
C.12	Significant difference between ratings for different occupations for LWFS, correlated with B.11, (*P* ≤ 0.05)
C.4	Difficult to verify, correlated with A.9
B.12	Very similar question to B.11, correlated with C.10
A.16	Very similar to factor A.1, correlated with B.7
B.10	Difficult to verify, requires specific skills, correlated with B.6
C.16	Difficult to verify on a single visit, same meaning as C.3 and correlated with B7
C.20	Very similar to factor C.22, correlated with B.6
D.10	Almost identical to C.17, correlated with A.9
D.9	Almost identical to B.1, correlated with A.9
C.8	Very difficult to verify on routine visit, correlated with B.11
C.7	Difficult to verify, significant difference between the rating for LWFS by occupation, correlated with B.7, (*P* ≤ 0.05)
D.3	Significant difference in ratings for LWFS, subjective and subject to officer’s opinion, correlated with B.6, (*P* ≤ 0.05)
A.2	Correlated with A.5
A.4	Correlated with A.9
A.8	Correlated with A.1
B.4	Correlated with A.1
C.11	Correlated with A.5
E.4	Correlated with A.1
E.33	Correlated with B.11

HWFS – high welfare farm survey

LWFS- low welfare farm survey

NT- Northern Territory, QLD- Queensland

The final list of risk factors retained to be included in the proposed AWRAT are reported in Table 2. The table includes the median ratings for the HWFS and LWFS for each factor and the H value from a Kruskal-Wallis test comparing ratings from participants with different occupations and locations. In the proposed AWRAT there was one factor that participants from different occupations had rated significantly differently (*P* = 0.047), this was ‘Investigation and treatment of health conditions affecting livestock are completed as soon as the problem is identified’. Veterinarians rated this factor (D.10), as more relevant than AWOs, who rated it as more relevant than stock agents, with median ratings of 7.5, 14 and 37, respectively. There was no significant difference between the ratings from participants from the north and south regions of Australia for the final 18 factors included in the proposed AWRAT.

**Table 2.** Risk factors to be included in the proposed AWRAT; median ratings for LWFS and HWFS; H value and significance of KWT comparing ratings for: LWFS and HWFS, participant occupations (LWFS) and location in north or south regions of Australia (LWFS)

Risk Factors	Median rating		KWT- comparing ratings from LWFS & HWFS	KWT- LWFS comparing ratings by participant occupations	KWT-LWFS comparing ratings from Nth/Sth Aus. regions
	LWFS	HWFS	H	H	H
A.1 – Farm fencing is of an effective standard (e.g. stock proof, swinging gates)	22	83	**57.59**	1.39	2.70
A.5 – The stock handling facilities are suitable and in working order	20	85	**75.52**	4.02	0.32
A.6 – Feed and water areas and facilities are clean, functional and accessible to all livestock	25	86	**78.04**	5.03	0.17
A.9 – The farm has an equivalent amount of pasture (or better) to that on surrounding farms	13	80	**67.80**	5.04	0.08
A.13 – Dead animals are removed from paddocks	22	78	**53.52**	5.53	0.32
B.1 – Livestock are fed according to their reproductive state (e.g. empty, pregnant or lactating)	16	88	**81.81**	0.22	0.22
B.6 – Some paddocks have good feed remaining	23	77	**58.47**	3.68	2.07
B.7 – Paddocks with livestock are suitable to avoid illness (e.g. toxic plants, feeding off the ground)	25	78	**59.97**	0.33	0.05
B.8 – There is some supplementary feed remaining in the paddock after the livestock have finished feeding	16	55*	**34.42**	3.98	0.05
B.9 – All animals have equal access to supplementary feeding (e.g. well spread out or continuous access)	23	74*	**54.99**	1.22	0.26
B.11 – Feed offered is suitable for the class of animal (e.g. energy, protein etc)	19	82	**70.03**	1.17	1.46
B.13 – Supplementary feeding is offered before significant weight loss occurs	11	80	**68.88**	7.64	0.40
D.1 – Lambs, calves and / or kids within a mob / herd are all a similar age (within 2–3 months of each other)	23	83	**64.36**	5.09	0.09
D.9 – Animals with conditions that are unresponsive or not economical to treat are humanely culled without delay (e.g. lameness, cancers, chronic scouring, congenital abnormalities)	19	86	**67.33**	6.06	0.86
D.10 – Investigation and treatment of health conditions affecting livestock are completed as soon as the problem is identified	1.5	85	**72.37**	**7.96**	0.26
D.17 – Livestock in poorer condition are drafted out of the mob/herd/ flock and preferentially fed	14	77	**51.85**	2.69	2.33
D.19 – The farm is stocked with the appropriate number of livestock for the area available (not overstocked)	20	90	**65.42**	3.41	1.17
D.22 – Entire male livestock are securely managed away from female stock that are too young and / or too small to be pregnant	22	95	**71.61**	6.63	0.75

KWT - Kruskal-Wallis test

LWFS – low welfare farm survey

HWFS – high welfare farm survey

H – test statistic for KWT

Nth- north, Sth – south, Aus. - Australia.

Bold text– significantly different (*P* ≤ 0.05)

* median ratings outside the key range (< 26, > 74)

## Discussion

One hundred and forty-one participants with some experience with extensively managed non-dairy cattle, sheep and goats, from several livestock-related occupations completed the risk factor survey. They provided ratings to indicate their view of the likelihood of observing each of 99 risk factors on farms with either high (HWFS) or low welfare (LWFS). Factors with ratings that were in the key range of < 26 and > 74 were considered potential risk factors of welfare or protective factors of welfare, depending on the question. For LWFS, there were 49 potential predictors of poor welfare with median ratings in the key range. The proposed animal welfare risk assessment tool (AWRAT) included 18 risk factors all of which are relatively easy to observe on a routine farm visit. This would enable the assessment to be completed without requiring more time or additional movement around the farm during a visit. This makes the tool both practical and feasible for officers to complete given that they are often restricted as regards time availability. Three of the risk factors related to poor infrastructure included issues with fencing, water and feed facilities, which have been reported by others previously (Väärikkälä *et al.* 2019; Williams *et al.* 2024). While the factor about removing dead animals from paddocks has been reported rarely (Williams *et al.* 2024), an increase in mortalities generally has been more frequently noted (Sandgren *et al.* 2009; Kelly *et al.* 2011). The proposed AWRAT included nine factors concerning nutrition, including the quantity, adequacy, suitability, management and accessibility of both pasture and supplementary feed. Inadequacy of nutrition in situations of poor welfare has been well reported previously (Kelly *et al.* 2011; Lomellini-Dereclenne *et al.* 2017; Väärikkälä *et al.* 2019, 2020). There was one factor about failing to provide adequate treatment, and this too is well documented on farms where there is poor welfare (Otten *et al.* 2014; Lomellini-Dereclenne *et al.* 2017; Väärikkälä *et al.* 2020; Williams *et al.* 2024). The last three factors, timely euthanasia, secure containment of males and the presence of similarly aged offspring, have been less frequently reported in the literature (Williams *et al.* 2024). The difference in risk factors identified in the literature and the proposed AWRAT may reflect the different production systems studied. As mentioned earlier, the majority of the literature is from Europe, where farming is more intensive with considerable over-winter housing of livestock compared to the predominantly extensive housing systems in Australia.

As the development of the proposed AWRAT was informed by experienced industry participants and based on Australian production systems, it is reasonable to suggest the risk factors will be reliable predictors of poor welfare in Australian pasture-based extensive farming conditions. Risk factors in risk assessment tools in other disciplines are selected using evidence-based research (Mason & Julian 2009; ACT Government 2022) as was used here. Although selection of factors for inclusion in the survey was robust, it is acknowledged that there will likely be other predictors of poor welfare that have been excluded or not considered here and potentially different risk factors that may be used to develop a risk assessment tool.

All factors related to management/husbandry and farmer characteristics failed to meet the criteria for inclusion in the proposed AWRAT. This was largely due to median ratings outside the key range. Some management factors were also removed because they were difficult to verify, were very similar to other factors or were not relevant to all extensive livestock farming systems in Australia. In particular, sheep-specific factors were removed as they are not relevant in all jurisdictions. The AWRAT is likely to still be effective at identifying sheep at risk of poor welfare based on other factors. In the Northern Territory, sheep are prohibited (Northern Territory Government 2022) and, in Queensland, only 3% of the Australian sheep and lamb population is farmed (Australian Bureau of Statistics [ABS] 2020). Despite considerable differences in farming systems between the north and south of Australia (Greenwood *et al.* 2018), there was no significant difference between the ratings of participants from LWFS for the two regions for any of the factors in the proposed AWRAT. This suggests that the same issues with infrastructure, nutrition and appropriate joining and treatment are relevant on properties with poor welfare, despite the vast differences in the type of extensive production system. In addition, there was no significant difference between the ratings for participants from different occupations for both surveys for all but one of the factors in the proposed AWRAT. Stock agents rated the factor about timely investigations and treatment of health conditions (D.10), as less relevant to low welfare farms than the other participants in this survey. This may indicate that stock agents have a differing perception of the impact of ill health or delayed treatment on animal welfare. As the overall median rating for this factor across all occupations was 14.5 and no other factors addressed this issue, it was retained in the proposed AWRAT.

The exclusion of farmer factors from the proposed AWRAT, based on not meeting the selection criteria, is both telling and appropriate. It is acknowledged that the farmer is crucial in determining the welfare outcomes of their livestock (Brumby *et al.* 2008; Andrade & Anneberg 2014; Coleman & Hemsworth 2014; Devitt *et al.* 2018). However, it could be argued that including factors about the farmer in a risk assessment tool is ethically flawed because of the personal nature of these factors. Alternatively, assessments focused on risk factors about the farm, nutrition and the animals, provide a measure of risk, based on the ‘circumstances’ in which the livestock are managed. The ‘circumstances’ also reflect upon the farmer’s behaviour, attitudes and capacity (Kelly *et al.* 2011; Andrade & Anneberg 2014; Devitt *et al.* 2018) without the need to consider the person directly.

While there is no single explanation for neglect of livestock (Andrade & Anneberg 2014; Devitt *et al.* 2015), the farmer is a crucial part of the solution (Kauppinen *et al.* 2010; FAWC 2016; Devitt *et al.* 2018). The human aspect of animal welfare requires more investigation and should be the focus of further research. A collaborative approach, where support for the farmer and the animals are both considered, is likely to be beneficial in establishing a change in behaviour to facilitate prompt and sustained welfare improvements (Kauppinen *et al.* 2010; Andrade & Anneberg 2014; Devitt *et al.* 2014, 2018; FAWC 2016).

As discussed previously, risk factors can be both predictors (Schooling & Jones 2018; Garrett & Monahan 2019) and a direct cause of negative welfare outcomes at the same time (Schooling & Jones 2018) or just predictors of an outcome. For example, the following factors from the proposed AWRAT can directly result in poor welfare: inappropriate water supply, inadequate nutrition and failing to provide prompt treatment or euthanasia for animals that are unwell, injured or recumbent. Other risk factors may contribute to the circumstances that make it more likely for animals to have poor welfare but are not a direct cause (Risvoll *et al.* 2017). Such factors include an absence of stock-proof fencing which means joining periods cannot be controlled, livestock cannot be fed preferentially, weaning is ineffective, and pasture and disease management is extremely difficult. Furthermore, poor infrastructure makes the performance of routine husbandry and management procedures very difficult or impossible. Some critical examples include shearing, crutching, marking, drenching, vaccinating and providing treatments. As the majority of the risk factors in the proposed AWRAT have a logical impact on welfare outcomes, it is proposed that they might also be used as the basis for discussion with the farmer regarding what is needed to be improved to reduce the risk of poor welfare. Through this engagement the challenges to making the required improvements may be identified and addressed (FAWC 2016; Devitt *et al.* 2018). Such challenges may include, but not be limited to, personal problems, financial pressure and health issues, as identified in previous literature (Andrade & Anneberg 2014; Devitt *et al.* 2015; FAWC 2016; Devitt *et al.* 2018).

Factors that are protective may buffer the effects of risk factors (Hoge *et al.* 1996; Rennie & Dolan 2010). They can also be useful to include in education and extension programmes (Stone *et al.* 2012) and are worthy of future consideration. Sixteen factors included in the proposed AWRAT were both risk and protective factors, with median ratings in the key range for LWFS and HWFS. The exceptions were the provision of supplementary feed with equal access (B.9) and surplus to immediate need (B.8). They were retained in the proposed AWRAT as they were easy to verify and met the other selection criteria. Participants also indicated they were more certain of factors that were present on high welfare farms, as 16 factors had median ratings in the top or bottom 10% of the scale, while no median ratings of factors in the LWFS were in that band. It is possible that the moderate values, outside the key range for LWFS, reflect participant uncertainty, possibly based on a relative lack of experience, rather than a definitive view that these factors were not relevant. For example, AWOs, arguably the most experienced in incidents of poor welfare in the LWFS, had 30% more median ratings in the key range than any of the other occupation responses in that survey.

A visual analogue scale (VAS) was used in this study as it is a simple and quick measurement tool for subjective phenomena (Wewers & Lowe 1990) and provides unrestricted opportunities to respond within the scale (Kuhlmann *et al.* 2017). Irregularities in ratings that have been observed by others were minimised by excluding tick marks and dynamic labelling on the slider thumb (Matejka *et al.* 2016). Some reports suggest that online surveys can result in score variation depending on the device used (Toepoel & Funke 2018) and systemic bias by precluding participation by some (Wright 2017). It has been suggested that face-to-face discussions generate a more representative sample of the socio-demographics of the population (Szolnoki & Hoffmann 2013), however this was not a reasonable option for this study.

There was a significantly greater number of ‘no responses’ for both LWFS and HWFS in the last section, compared to previous ones. Similarly, others have reported an increase in the number of ‘no response’ (Krosnick *et al.* 2002) and ‘don’t know’ responses towards the end of surveys (Deutskens *et al.* 2004). In the last section, only 18% of the median ratings were in the key range in the LWFS, significantly lower than the other sections. It is possible this may be due to declining motivation, which has been associated with responding to questions in the easiest possible way, for example, providing similar responses (Herzog & Bachman 1981; Galesic & Bosnjak 2009) or ratings that are less definitive (Deutskens *et al.* 2004). Missing values are recognised as a disadvantage of online surveys (dell’Olio *et al*. 2018). It would have been beneficial to randomly select the order of the survey’s subsections in order to ensure different respondents completed different sections last (Herzog & Bachman 1981), thereby mitigating the impact of declining motivation.

The study had some limitations, including the number of responses without ratings, the relatively small sample of participants and the uneven representation across the jurisdictions. The response rate was low considering the large number of engagement emails sent. This may have been due to the impersonal nature of the engagement email and an increasing hesitance to use links from unfamiliar email addresses. The poor response rate may have resulted in response bias, where participants interested in the topic or more comfortable with internet use were more likely to respond (dell’Olio *et al.* 2018). Lastly, it was not possible to validate the survey participants’ interpretation of what constituted good or poor animal welfare. Engaging survey participants that had experience working in the livestock industry and providing a definition of animal welfare, was intended to minimise this uncertainty. Overall, these challenges were unlikely to reduce the robustness of the risk factors selected, as long as they are shown to be correlated with incidents of poor livestock welfare in a validation trial (Garrett & Monahan 2019).

Further work is necessary to test and verify the effectiveness of the proposed AWRAT to correctly predict livestock welfare outcomes. A trial will include assessments on farms visited for the investigation of poor welfare and others for non-welfare related reasons. This will allow the predictive capacity of the proposed AWRAT to be tested on properties with different welfare standards, so the results can be compared. A standard operating procedure would provide detailed explanations of the factors and how they can be assessed on-farm to ensure all participants in the trial have the same understanding on how to use the tool. Then appropriate scoring and weighting will be determined (van Ginneken 2019) to ensure the tool has the best predictive capacity. The quality of the data will be crucial to ensure a reliable assessment (Gottfredson & Moriarty 2006).

An Animal Welfare Risk Assessment Tool (AWRAT) is likely to be of most value in identifying farms where the livestock are at risk of poor welfare. If farms ‘at risk’ with a low standard of welfare but not in breach of the legislation could be identified early, intervention and extension could be employed to protect the animals from any further decline in welfare. This would also reduce the resources required to respond and reduce the loss of production and income that would affect the farmer. Animal welfare response agencies might use an AWRAT assessment to prioritise cases and inform the allocation of resources, the frequency of revisits, the use of legal instruments, potential prosecution and ongoing surveillance. The risk assessment could also provide a discussion point with the farmer to identify the issues that need to be addressed to decrease the risk of poor welfare. In ongoing situations that are challenging to resolve, repeat AWRAT assessment would provide an objective way of communicating the lack of progress in reducing the risk (Zsidisin *et al.* 2004). Potentially, in cases found guilty of cruelty in court, the proposed AWRAT may be presented to the judge to demonstrate the risk of reoffending, this may be used to inform sentencing, as reported in other disciplines (Kleiman *et al.* 2007; van Ginneken 2019). Others warn, though, for a risk assessment to be used at sentencing it needs to be publicly transparent, scrutinised by scientists, technically and statistically valid and valued by those involved in decision-making (Garrett & Monahan 2019).

### Animal welfare implications

The development of an animal welfare risk assessment tool (AWRAT) may allow livestock at risk of poor welfare to be identified. This would facilitate early intervention, extension and education to improve the care, health and welfare of the livestock. Potentially, an AWRAT may provide a structured format to discuss with the farmer what needs to be improved and provide a way to monitor those improvements. In court, an AWRAT assessment might be used to inform the judge of the likelihood of reoffending in cases prosecuted under animal welfare legislation, and this might inform sentencing.

## Conclusion

Participants agreed that 49 risk factors were commonly observed on properties where livestock welfare is poor. This was reduced to a final list of 18 factors that have been used to develop a proposed AWRAT. The predictive capacity of the proposed AWRAT will need to be tested by performing assessments on farms with different welfare standards, in a future study.

## Supporting information

Williams et al. supplementary material Williams et al. supplementary material

## Supplementary material

The supplementary material for this article can be found at http://doi.org/10.1017/awf.2024.27.
