# Supplementary material for: Are there risk factors commonly observed on Australian farms where the welfare of livestock is poor?
Source: Anim Welf. 2024 Sep 16;33:e34. doi: 10.1017/awf.2024.27 (PMC11418073; doi:10.1017/awf.2024.27)
Supplement: Williams et al. supplementary material [file S0962728624000277sup001.pdf]

Factors included in the survey by category.

Section A: Farm factors

**Section A: Farm Factors**
A.1 - Farm fencing is of an effective standard (e.g. stock proof, swinging gates)
A.2 - Farm weeds appear controlled
A.3 - Farm driving tracks are in good condition (e.g. not overgrown, minimal pot holes)
A.4 - The farm is neat and tidy
A.5 - The stock handling facilities are suitable and in working order
A.6 - Feed and water areas and facilities are clean, functional and accessible to all livestock
A.7 - The farm has other enterprises, for example: cropping, commercial hay production, chickens, pigs
A.8 - There are signs of new or improved infrastructure: yards, fences, shed, machinery
A.9 - The farm has an equivalent amount of pasture (or better) to that on surrounding farms
A.10 - The farmer owns or have access to more than one block of land or farm (e.g. leased, agisted or owned)
A.11 - Livestock have access to shelter / protection from heat and cold
A.12 - Water is available at all times (not reliant on cartage)
A.13 - Dead animals are removed from paddocks
A.14 - Livestock grazing areas are suitable to protect against accidental injury (e.g. loose wire, debris, fences etc.)
A.15 - More than one animal type is kept in the same paddock (e.g. sheep, goat’s cattle, horse, pigs)
A.16 - The animals are securely contained by adequate perimeter fences

Section B: Nutrition Factors

**Section B: Nutrition Factors**
B.1 - Livestock are fed according to their reproductive state (e.g. empty, pregnant or lactating)
B.2 - The livestock approach vehicles that are in or near the paddock (suggesting they are used to being supplementary fed)
B.3 - There is hay, grain or pellets stored on the property
B.4 - The hay, if present, has good nutritional value (e.g. not weather affected or poor quality)
B.5 - Paddocks are grazed on rotation
B.6 - Some paddocks have good feed remaining

B.7 - Paddocks with livestock are suitable to avoid illness (e.g. toxic plants, feeding off the ground)
B.8 - There is some supplementary feed remaining in the paddock after the livestock have finished feeding
B.9 - All animals have equal access to supplementary feeding (e.g. well spread out or continuous access)
B.10 - Some of the paddocks have improved pasture
B.11 - Feed offered is suitable for the class of animal (e.g. energy, protein etc)
B.12 - Enough feed is provided (suitable quantity to meet requirements)
B.13 - Supplementary feeding is offered before significant weight loss occurs

Section C: Management and Husbandry Factors

**Section C: Management and Husbandry Factors**
C.1 - The livestock appear content, often lying down, chewing their cud
C.2 - The farm business includes breeding livestock
C.3 - There are no more livestock carcasses on the property than would be expected
C.4 - Sheep do not have over grown wool and / or are overdue for crutching (lots of dags)
C.5 - There are no livestock scouring
C.6 - Most of the livestock are in condition score 2-3 out of 5
C.7 - The males (rams, bulls or bucks) are in kept in good condition
C.8 - The animals do not seem unwell, lame or injured (e.g. animals are not away from the mob / herd alone or are falling to the back of the mob)
C.9 - Livestock are kept in groups according to their production status (e.g. dry animal, lactating animals, weaners)
C.10 - The farmer identifies livestock in poorer condition and drafts them out for preferential feeding
C.11 - Livestock that are found to be recumbent are euthanised or treated on the same day
C.12 - All livestock are in condition score 1.5 / 5 or more
C.13 - Livestock are treated effectively for lice (e.g. no scratching or rubbing, no wool on fences and no bald and warn areas on the animal's coat / wool)
C.14 - Condition scoring is used to monitor the condition of livestock

Section D: Animal Factors

**Section D: Animal Factors**
D.1 - Lambs, calves and / or kids within a mob / herd are all a similar age (within 2-3 months of each other)
D.2 - Lambs, calves and / or kids are marked when they are small (before 12 weeks of age)
D.3 - Offspring are weaned from breeding females prior to the next calving, kidding or lambing season
D.4 - The farm has a worm control program (e.g. faecal egg counts, drenching plan)
D.5 - Shearing is completed at least annually
D.6 - Crutching is completed in a timely manner (to mitigate fly strike risk)
D.7 - Livestock that are lambing, calving and /or kidding are suitably supervised (at least once daily)
D.8 - Livestock are mouthed and broken-mouthed animals culled or managed preferentially
D.9 - Animals with conditions that are unresponsive or not economical to treat are humanely culled without delay (e.g. lameness, cancers, chronic scouring, congenital abnormalities)
D.10 - Investigation and treatment of health conditions affecting livestock are completed as soon as the problem is identified
D.11 - All livestock are checked at least every second day
D.12 - Breeding aged females are joined for a set period of 5-12 weeks
D.13 - The farmer has the ability to euthanise animals appropriately and in a timely manner
D.14 - Fly strike prevention and treatment strategies are suitable (e.g. crutching, jetting, timely treatment of strike, euthanasia where required)
D.15 - All animals in a mob are shorn at the same time
D.16 - Breeding females have a break from lactating between weaning last season's offspring and lambing, kidding or calving again
D.17 - Livestock in poorer condition are drafted out of the mob/herd/ flock and preferentially fed
D.18 - Animals are marked in a manner that minimises the impact on their health and welfare
D.19 - The farm is stocked with the appropriate number of livestock for the area available (not overstocked)
D.20 - Only females of a suitable body condition and size are joined
D.21 - In breeding production systems, some livestock are sold off the farm regularly (at least annually)
D.22 - Entire male livestock are securely managed away from female stock that are too young and /or too small to be pregnant
D.23 - Shearing occurs at a time of the year that minimises the risk of cold and / or wet weather

Section E: Farmer factors

**Section E: Farmer factors**
E.1 - The welfare of the farmer's livestock is always good
E.2 - The farmer seems happy and content
E.3 - If you visit unannounced, the farmer is usually in the house
E.4 - The farmer has some formal training and / or a qualification in livestock farming
E.5 - Others in the farmers family have livestock with good welfare (e.g. parents, siblings, children, uncle or aunty)
E.6 - The farmer is NOT involved in a dispute (e.g. marriage break down, neighbourly or family dispute)
E.7 - The farmer is an absentee farmer (lives at an address that is not at any of their farming properties, e.g. in the city or in town)
E.8 - The farmer works hard on the farm
E.9 - The farmer has a perceived or real shortage of time (e.g. always in a hurry, hard to catch)
E.10 - The farmer has an apparent or perceived physical health condition that impedes their ability to perform farm duties consistently
E.11 - The farmer has an apparent or perceived mental health condition that impedes their ability to perform farm duties consistently
E.12 - The farmer is always quick to act and get jobs done
E.13 - When you visit, recent farmer activities are evident (e.g. feeding out, fences fixed, recent tyre marks in paddocks)
E.14 - The farmer enjoys livestock farming
E.15 - The farmer agrees with your description of the condition of the animals (e.g. skinny, fat or other)
E.16 - The farmer takes responsibility for the condition of their livestock
E.17 - The farmer also works off the farm
E.18 - The farmer has significant commitments and responsibility apart from the farm
E.19 - The farmer has an excellent knowledge of basic livestock management
E.20 - The farmer is able to adapt to changing conditions and new challenges
E.21 - The farmer's age does not negatively impact on their ability to farm
E.22 - The farmer is easy to get along with
E.23 - The farmer cares about the condition of their livestock
E.24 - The farmer is financially secure
E.25 - The farmer has support from family and / or friends

E.26 - The farmer follows advice and instructions
E.27 - The farmer understands their role and responsibility to provide care for their livestock
E.28 - The farmer has access to assistance on the farm to complete essential tasks if required
E.29 - The farmer has no behaviours or challenges that make it difficult for them to farm effectively
E.30 - The farmer has multiple properties
E.31 - There is an obvious ‘extenuating circumstance’ that explains any poor livestock welfare situation
E.32 - The farmer is honest and reliable
E.33 - The farmer employs a manager
